# Noise-Induced Hearing Loss: Updates on Molecular Targets and Potential Interventions

**DOI:** 10.1155/2021/4784385

**Published:** 2021-07-06

**Authors:** Huanyu Mao, Yan Chen

**Affiliations:** ^1^ENT Institute and Department of Otorhinolaryngology, Eye & ENT Hospital, Fudan University, Shanghai 200031, China; ^2^NHC Key Laboratory of Hearing Medicine (Fudan University), Shanghai 200031, China

## Abstract

Noise overexposure leads to hair cell loss, synaptic ribbon reduction, and auditory nerve deterioration, resulting in transient or permanent hearing loss depending on the exposure severity. Oxidative stress, inflammation, calcium overload, glutamate excitotoxicity, and energy metabolism disturbance are the main contributors to noise-induced hearing loss (NIHL) up to now. Gene variations are also identified as NIHL related. Glucocorticoid is the only approved medication for NIHL treatment. New pharmaceuticals targeting oxidative stress, inflammation, or noise-induced neuropathy are emerging, highlighted by the nanoparticle-based drug delivery system. Given the complexity of the pathogenesis behind NIHL, deeper and more comprehensive studies still need to be fulfilled.

## 1. Introduction

Hair cells (HCs) in the inner ear cochlea function in transducing sound waves into electric signals [1–4], while supporting cells function in supporting the HCs and providing the potential pool for HC regeneration [5–9]. Damages from a variety of sources can impair HC function, including mutations in deafness genes, aging, ototoxic drugs, chronic cochlear infections, and noise exposure [10–13]. Acoustic overexposure would result in sensorineural hearing loss characterized by high-frequency hearing threshold shift mainly, termed as noise-induced hearing loss (NIHL)[14], which is due to loss or damage of sensory HCs and degeneration of the spiral ganglion neurons (SGNs). Though noise susceptibility is quite individual, it is normally acknowledged that noise above 85 dB would be considered as hearing harmful [15, 16]. Studies [17–19] have shown that mild or moderate noise would only trigger temporary hearing threshold shift (TTS), because the noise-induced hair cell damage and auditory nerve fiber degeneration were still reversible. Hearing ability is commonly measured by auditory brainstem response (ABR) and distortion product otoacoustic emission (DPOAE) test [20]. The ABR threshold and DPOAE level would recover to prenoise level over time in TTS patients. However, severe noise exposure, referring as strong sound vibrations, or longtime exposure to harmful noise, or both, can lead to cochlear hair cell necrosis and apoptosis, causing permanent threshold shift (PTS)[16, 21]. Although the neonatal mammals still have very limited HC regeneration ability, the adults have lost this ability [22–26], leading to the irreversible loss of HCs after noise damage. Besides decrease of auditory sensitivity and language recognition ability, NIHL patients could suffer from headache, tinnitus, dizziness, hypertension, etc. [14, 17].

It is estimated that about 5% of the world's population suffer from disabling hearing loss [27]. Modern people are exposed to acoustic trauma with increasingly higher risk as the industrialization of society. Strikingly, evidence has shown that acoustic trauma-induced hearing impairment aggravates microglial deterioration around the hippocampus, indicating its potential causal role in the pathogenesis of dementia, which exacerbates the already overloading public health burden of NIHL [28]. Therefore, more and more attention has been focused on revealing the pathogenesis mechanisms and effective therapeutics of NIHL. We review the updates on molecular targets about NIHL, summarize the approved and potential medications for NIHL treatment, and propose the controversies and foreseeing on NIHL.

## 2. Cellular Pathologies

Acoustic shock with strong sonic energy caused temporary or permanent cellular pathologies. Hair cells, especially outer hair cells (OHCs), are the main targets of noise trauma. Noise overexposure causes hair cells swelling and eventual irreversible death [29]. Noise-induced sensory hair cell impairment also accompanies by collapse and loss of stereocilia and destruction of their tip connection. Interestingly, the loss of tip links connecting adjacent stereocilia would increase noise susceptibility reversely [30–32]. Acoustic overstimulation can also cause the reduction of synaptic connections between hair cells and spiral ganglion cells, afferent fiber swelling, and auditory nerve deterioration [33–37].

It is widely recognized that noise with sound intensity > 85 dB is hearing harmful, while extremely high intensity noise like blast or gunshot could do much more harm to the hearing system within very short exposing time. The latter has been given a special name termed blast-induced hearing loss to highlight its difference from chronic NIHL [38]. Besides the damage of hair cells and spiral ganglion neurons, high intensity noise can result in middle ear damage like tympanic membrane perforation, ossicular chain dislocation, oval/round window rupture, and thus external lymph fistula [39]. The stria vascularis and spiral ligament are also attacked by intense noise, resulting in lower blood flow and lower vessel diameter in the stria vascularis [40]. Hearing impairment is the foremost clinical symptom of blast overexposure injury. Besides that, blast may also cause otitis media because of the rupture of tympanic membrane and secondary infection [41]. Vertigo and balance disorder occur in patients accompanied by labyrinthine damage and hemorrhage after blast exposure, which leaves headache and dizziness for a long time after the accident. Therefore, the treatment of blast-induced hearing loss often involves operations like tympanoplasty to repair tympanic membrane and rebuild ossicular chain [42].

## 3. Molecular Pathogenesis

**Figure 1:**
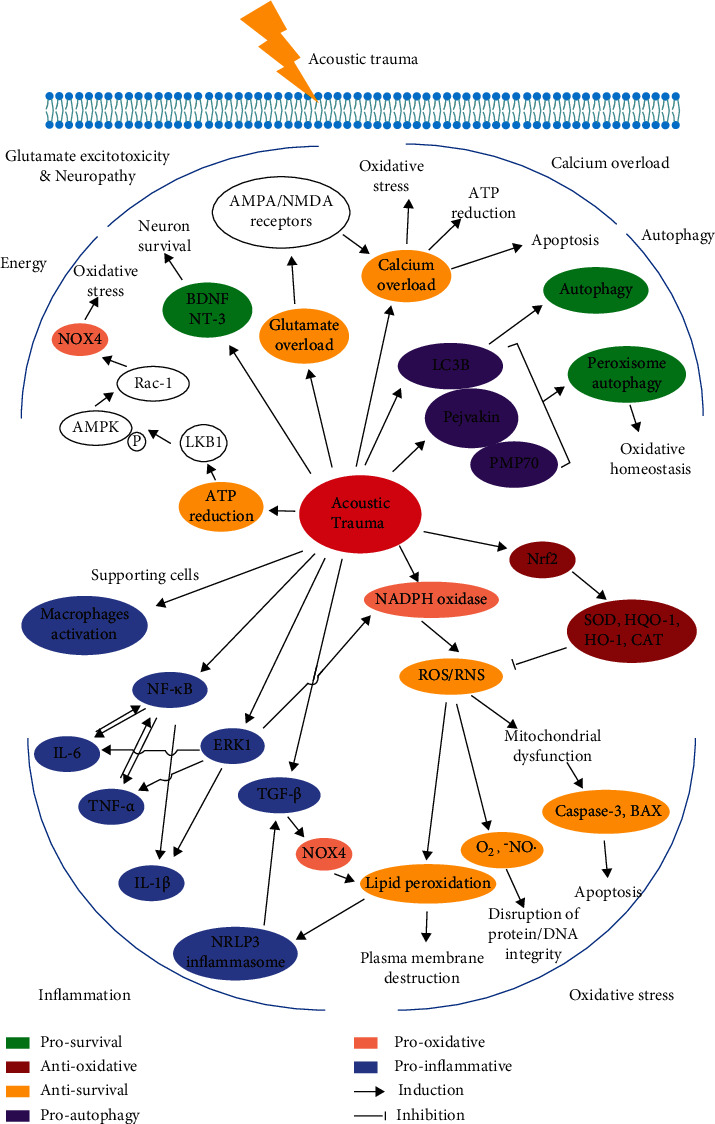
The molecular pathogenesis of NIHL.

Acoustic trauma features hair cell loss, synaptic ribbon deterioration, and acoustic nerve degeneration. Previous studies [43, 44] have proved that after noise exposure, both necrosis and apoptosis occurred in the sensory epithelium. However, the causative mechanisms inducing necrosis and apoptosis are much complex and intertwined (Figure 1).

### 3.1. Oxidative Stress

Though the exact mechanism behind NIHL is not fully explained yet, various studies [45–47] have suggested that oxidative stress in the cochlea contributes to the noise-induced hearing impairment. Ohlemiller et al. [45] have proved that the production of hydroxyl radical, a main kind of reactive oxygen species (ROS), increases over four times upon acoustic trauma in cochlear perilymph. ROS, mainly generated from the leaking electrons in the mitochondrial electron transport chain, is crucial for multiple physiological behaviors like cell proliferation and differentiation in relative low content [46, 48]. Under physiological circumstances, the production and scavenging of ROS are maintained at balance subtly and dynamically. Many stress stimulators like extensive sound exposure as well as ototoxic drugs like cisplatin [47] and aminoglycosides [49–56] can increase ROS production in hair cells, which is beyond the maximal cellular antioxidative ability, and threaten the integrity of DNA, protein, and other survival-crucial macromolecules [57]. Similar to ROS, reactive nitrogen species (RNS) also contribute to the noise-induced cochlear oxidative imbalance [58]. Overproduction of ROS/RNS in hair cells and spiral ganglion neurons is well studied [59, 60], whereas the role of oxidative stress in supporting cells still needs further explanation. Styrene was applied to induce specifically oxidative damage in cochlear supporting cells, and hearing ability was also apparently impaired [61]. However, how oxidative stress in supporting cells participates in NIHL progression requires deeper exploration.

The production and scavenging of ROS/RNS are mediated by various endogenous antioxidants and antioxidative enzymes [57]. The enzymes producing ROS/RNS are usually kept at low expressing level in hypermetabolic cells like acoustic hair cells, tumor cells, and cardiac myocytes, which are oxidative stress sensitive. The NADPH oxidase family (NOX) has been proved to catalyze the electron transport from NADPH to oxygen molecules to speed up ROS production [62]. The *NOX4* transgenic mice which constitutively expressed human *NOX4* were found to be noise susceptible while they had normal hearing threshold without acoustic stimulation, stressing the vital role of imbalance of redox homeostasis in NIHL [63].

Besides ROS/RNS overproduction, the inducible transient upregulation of antioxidative genes also features the pathogenesis of NIHL as a physical feedback mechanism. Nrf2, which is widely expressed in multiple tissues such as the heart, liver, and cochlea, can respond to oxidative stress and activate downstream antioxidative gene expression like glutathione peroxidase (GPx), NAD(P)H:quinone oxidoreductase 1 (NQO1), heme oxygenase-1 (HO-1), and catalase (CAT) [64–66]. Nrf2 signaling can also activate autophagy in hair cells via p62 protein for oxidative stress amelioration [67]. More and more researches [67, 68] have proved that targeting Nrf2 signaling is a promising therapeutic solution for NIHL.

Given the fact that oxidative stress is one of the key contributors to the pathogenesis of NIHL, it is reasonable to assume that genes involved in the redox homeostasis correlate to noise vulnerability of the cochlea [69]. Superoxide dismutase (SOD) and paraoxonases (PONs) are two antioxidative enzymes found in the cochlea; the former directly catalyzes superoxide radicals to less toxic hydrogen peroxide while the latter decreases lipid peroxidation activity [70]. It is reported that the polymorphisms of SOD2, PON1, and PON2 are related to susceptibility to NIHL in workers with high occupational noise trauma risk [69, 71]. Meanwhile, overexpression of SOD1 was found to provide a protective effect on noise-related hearing loss [72]. Another newly emerging medication named nicotinamide, the NAD^+^ precursor, can boost NAD^+^ content and ameliorate NIHL in a SIRT3-dependent way [73]. SIRT3 plays an important role in maintaining the function of various antioxidative enzymes while impaired SIRT3 resulted in ROS overproduction and decreased levels of GSH [74, 75].

### 3.2. Autophagy

Autophagy, as a recently found endogenous self-defense mechanism, degrades damaged organelles by lysosome to maintain internal homeostasis and preserve energy providing for cell survival upon stress like nutrient deficiency, pathogen infection, protein misfolding, and oxidative stress [76–80]. Previous studies [81, 82] have shown that autophagy is upregulated in auditory hair cells after intense noise exposure. The immunostaining results show the upregulation of LC3b, the indispensable component of autophagosome, and the colocalization of LC3b and Lamp1, indicating the enhanced fusion of autophagosome and lysosome. To test the hypothesis of autophagy as the cochlear endogenous defenses against NIHL, researchers analyzed whether the disruption of autophagy could exacerbate NIHL with autophagy direct inhibitor 3-MA and mTOR signaling agonist RAP. mTOR protein is one of the main negative regulators for autophagy induction because it can phosphorylate Atg13 to cut off the beginning phase of autophagosome formation [83]. Notably, the inhibition of autophagy worsened the noise-induced oxidative imbalance and the eventual hair cell loss and impaired hearing ability [81].

Pejvakin protein is recently identified as the oxidative sensor in auditory hair cells. Researchers applied immunostaining colocalization analysis and in situ PLA technology to testify the enhanced interactions between pejvakin and LC3B, as well as pejvakin and PMP70 (the marker of peroxisome) in hair cells after acoustic trauma or simply H_2_O_2_ treatment. Furthermore, pejvakin is found to mediate peroxisome autophagy after noise exposure, which is vital to secondary peroxisome proliferation and oxidative homeostasis maintenance afterwards. Pejvakin-mutated mice show impaired peroxisome proliferation and more severe oxidative stress in auditory hair cells after noise exposure [84].

### 3.3. Inflammation

The cochlea was used to be considered as immune exemption organ because of the blood-perilymph barrier [85]. However back in 2008, Okano et al. [86] transplanted the EGFP-positive hematopoietic stem cells (HSCs) into wide-type C57BL/6 mice to label the bone marrow-derived cells (BMDCs) with green fluorescence and found EGFP-positive cells in cochlear spiral ganglion and spiral ligament regions of the HSC transplanted mice. Interestingly, EGFP-positive cells were colocalized with F4/80, Iba1, and CD68 immunostaining marks, indicating the presence of bone marrow-derived cochlear resident macrophage-like cells. Like microglia in the nervous system, supporting cells can also be stained with various macrophage markers surprisingly. LPS treatment can induce phagocytosis of supporting cells, which is the direct functional evidence linking cochlear supporting cells with macrophages [87]. Recently, more and more researches suggest the key role of inflammation in aging, drug, and noise-induced ototoxicity [10]. It is notable to point out that inflammation is a dual sword that requires a very complicated and delicate regulatory network involving multiple genes and transcription factors. After acoustic trauma, inflammatory cells representing CD45, CD68, and Iba1 positive infiltrate and proliferate in the cochlea, especially in the stria vascularis, with high expression of CX3CR1, the receptor of a classic chemokine [88, 89]. These inflammatory cells can synthesize and release a variety of proinflammatory cytokines like interleukin-1*β* (IL-1*β*), interleukin-6 (IL-6), and tumor necrosis factor-*α* (TNF-*α*)[90]. IL-1*β* and IL-6, the markers of acute inflammation belonging to the interleukin superfamily, contribute to the mononuclear phagocyte infiltration and subsequent release of elastase and prostaglandin, bringing to excessive inflammation cascade activation resulting in microcirculation disturbance, tissue destruction, and eventual hearing impairment [88, 89, 91].

Targeting TNF-*α* inhibition by molecules or siRNA also attenuates the noise-induced cochlear inflammation and thus hearing threshold shift [92–94]. It is reported that noise-induced increase of TNF-*α* can be regulated by nuclear factor of activated T cells 4 (Nfatc4), a well-researched transcription factor served as proinflammatory cytokine inducer. Nfatc4 knockout mice show resistance to TNF-*α*-mediated hair cell apoptosis triggered by intense noise [95]. Notably, TNF-*α* is testified to be able to induce calcium influx through ERK-dependent activation of TRPV1. Desensitization of TRPV1 by capsaicin ameliorates TNF-*α*-induced calcium influx and hair cell apoptosis in NIHL modeling mice [96].

Tumor growth factor-*β* (TGF-*β*) has been proved to be pluri-regulatory in cochlear development and homeostasis maintenance such as otic capsule formation [97], spiral ganglion neuron survival [98, 99], and inflammation responses [100]. TGF-*β* initially serves as the inducer of adhesion and chemoattractive molecules for the chemotaxis and activation of leukocytes, and downregulates the interleukin production reversely as the inflammation progresses, indicating its double effects on inflammation regulation [101–103]. Reportedly, the mRNA expression level of TGF-*β* is upregulated in the cochleae upon noise trauma. Using P7 and P144, two peptides targeting TGF-*β* inhibition, significantly improves hearing compared to the control group in NIHL mouse model [104]. Further evidence indicates that TGF-*β* inhibitor decreased the NOX4 content, which is proved to promote NLRP3 inflammasome activation by oxidating fatty acids [105].

It is well testified that nuclear factor kappa B (NF-*κ*B) and its target proinflammatory genes play the central roles in inflammation regulation. Previous researches [94, 106, 107] have found that NF-*κ*B signaling cascade can be activated by noise trauma and inhibition of NF-*κ*B can decrease the content of proinflammatory cytokines. Glucocorticoid is the most commonly used medication for the treatment of NIHL, and its pharmaceutical activity is reported to be partly mediated by NF-*κ*B inhibition [108]. Another interesting discovery about NIHL is that mice show greater sensitivity to noise overexposure during the night [109]. The circadian sensitivity alteration of the cochlea is proved to be adrenal gland-derived endogenous glucocorticoid dependent for its key role on inflammation regulation [110]. Besides, proinflammatory meditators like cyclooxygenase-2 (COX2) and NADPH oxidase 3 (NOX3) are also found upregulated after noise exposure, verifying the indispensable role of inflammation in the pathogenesis of NIHL [94].

### 3.4. Glutamate Excitotoxicity and Neuropathy

The acute cochlear synaptic ribbon disruption and lasting acoustic neural degeneration also underlie the NIHL pathogenesis. Studies [111, 112] have proved that glutamate excessive accumulation serves as the contributor to acute and chronic deterioration of the auditory nervous system. Glutamate is subtly regulated and remains at a very low level in the synaptic cleft. The overloading of glutamate might dysregulate the cystine-glutamate transporter and result in cystine and thus GSH deficiency, or constantly activate the glutamate receptor in the postsynaptic membrane and damage the afferent neural fibers [113, 114]. GLAST, one of the five classic glutamate transporters, is found to be expressed in supporting cells located around the inner hair cells. GLAST plays a crucial role in glutamate uptake for its rapid recirculation in the synaptic cleft between the inner hair cells and afferent auditory neural fibers, and depletion of GLAST leads to excessive accumulation of glutamate, which might trigger calcium overload in spiral ganglion neurons. The knockout of GLAST exacerbates the hearing loss in transgenic mice, indicating the potential role of glutamate excitotoxicity in NIHL [111]. The release of glutamate from inner hair cells is also vesicular glutamate transporter type-3 (vGlut3) dependent. Despite the fact that glutamate excitotoxicity relates to NIHL pathogenesis, glutamate also has its dual effects on the noise-induced synapses loss. vGlut3-KO mice show moderate to severe hearing loss due to the impaired synaptic connection development for IHCs and acoustic nerve fibers. And Vglut3^+/-^ mice show disrupted synaptic ribbon recovery after acoustic trauma, indicating that glutamate also participates in the repair of ribbon synapses in TTS model of NIHL [112].

Previous studies [115, 116] have suggested that calcium overload as a vital contributor to glutamate excitotoxicity. There are two main kinds of glutamate receptors in the postsynaptic region, termed as N-methyl-d-aspartate (NMDA) receptor and *α*-amino-3-hydroxy-5-methyl-4-isoxazole-propionate (AMPA) receptor [117]. Though NMDA receptors play the key role in calcium influx and consequent excitotoxicity in the central nervous system, intratympanic injection of NMDA receptor antagonist AM-101 shows no obvious protective effect on the tone ABR threshold at any frequencies, indicating that calcium influx mediated by NMDA receptors is not the most important contributor of glutamate excitotoxicity in the cochlea [115].

As for AMPA receptor, it is an ionic channel type receptor of glutamate, containing four subunits, namely, GluA1, GluA2, GluA3, and GluA4. Once activated, AMPA receptors mediate fast moving of multiple ions crossing cellular membrane [117, 118]. GluA2 has been testified to decrease Ca2^+^ permeability greatly [119–121]. Recent studies have revealed the existence of GluA2-lacking calcium-permeable AMPA receptors (CP-AMPARs) [116, 122]. Interestingly, IEM-1460, a CP-AMPAR inhibitor, significantly ameliorates the noise-induced decline of synapse integrity, as well as ABR threshold shift, shedding light on the therapeutic potential of NIHL by targeting CP-AMPARs [116].

The reduction of synaptic ribbons and acoustic neural degeneration is reversible in noise-induced transient hearing loss. It is reported that the recovery of cochlear synaptic ribbons after noise exposure is regulated by some endogenous neural factors named neurotrophins. Neurotrophins are endogenous molecules which are vital to the morphogenesis of nervous system development and neuron survival. Brain-derived neurotrophic factor (BDNF) and neurotrophin-3 (NT-3) are highly expressed in the inner hair cells and supporting cells to guide the auditory neural fibers to form precise synaptic connection in postnatal mice and stabilize the connection in adult mice [123]. Knockout of Ntf3 in supporting cells using Plp1-CreER mouse line impairs the high-frequency hearing while Bdnf depletion does not. Reversely, overexpression of Ntf3 or Bdnf in supporting cells and hair cells both attenuate the noise-induced synaptic ribbon reduction and eventual hearing loss [124]. Direct administration of NT-3 also attenuates NIHL progression [125]. Interestingly, as the detection technology develops into more microcosmic level, NT-3 is also found in the extracellular vesicles from human multipotent stromal cells (MSC-EVs). And administration of MSC-EVs not only raises the survival rate of spiral ganglion neurons cultured *in vitro* but also ameliorates the NIHL *in vivo* [126].

The effect of BDNF on NIHL is quite complicated and controversial. Many studies have reported that BDNF administration can enhance auditory neuron survival [127–129]. Meanwhile, tropomyosin-related kinase type B (TrkB, a selective BDNF receptor) agonist DHF [109], amitriptyline, and 7,8-dihydroxyflavone [130] increase resistance to acoustic trauma. However, genetically, depletion of Bdnf gene in adult mice shows protective effect against NIHL because of impaired glutamate release from IHCs, indicating the essential role of BDNF for maintenance of IHC function as well [131].

### 3.5. Calcium Overload

Calcium is one of the most important ions for the development and physiological function of the inner ear [132, 133]. Under physiological circumstances, calcium content in hair cells and spiral ganglion neurons is quite low [134, 135]. In response to the sound stimulation, calcium influx and calcium-induced calcium release (CICR) from the endoplasmic reticulum significantly increase intracellular calcium and trigger the release of neurotransmitter from the IHCs to transform mechanical signals to electrical signals [136, 137]. Moreover, the spontaneous calcium spike from the IHCs also participates in the formation and functional maturation of the afferent auditory nerve [138]. Calcium takes part in various physiological functions as one of the main second messenger molecules, while excessive accumulation of calcium under stress like ischemia-reperfusion injury [139] contributes to cell death in several pathways. Previous studies show that calcium is also involved in the acoustic ototoxicity [135, 140, 141]. Intracellular calcium concentration is significantly upregulated in auditory hair cells, especially outer hair cells shortly after acoustic overstimulation though returns to basal level within 30-45 minutes [135]. The calcium concentration is also found increased abruptly in endolymph of the cochlea after intense noise exposure [141]. Minami et al. have reported that the expression of calcineurin, a calcium/calmodulin-dependent phosphatase, is restrictedly increased in dying hair cells marked by propidium iodide after noise overstimulation [142]. Calcineurin can dephosphorylate and activate the classic proapoptotic protein BAD, and the systemic administration of calcineurin inhibitor FK506 reduced the apoptosis of hair cells evidently [142, 143].

Though strong evidence has proved that excessive calcium overload contributes to noise-induced hair cell death, the pharmacological drug development targeting calcium overload is still contentious. Voltage-gated calcium channels (VGCCs) facilitating calcium influx are proved to be responsible for the noise-induced intracellular calcium overload. There are five different types of VGCCs, namely, L-, T-, N-, P/Q-, and R-type [144]. It is reported that administration of T-type calcium channel blockers like ethosuximide and trimethadione prior to noise exposure can effectively reduce the hearing threshold shift [145]. However, other researchers suggest that L-type calcium channel blockers [146] attenuate NIHL while T-type calcium channel blockers cannot.

### 3.6. Energy Metabolism

Transient ATP depletion in the cochlear perilymph following acoustic trauma has been proved by studies [147, 148]. Likewise, the extracellular ATP content in the inner ear also decreased upon ischemia stress [149]. Since either the production of endogenous antioxidants or the recovery of spiral ganglion cells upon noise exposure is energy consuming, researchers proposed the hypothesis that transient ATP depletion might weaken the defense ability of the cochlea against acoustic trauma. 5′-Adenosine monophosphate- (AMP-) activated protein kinase (AMPK), the energy sensor protein responding to fluctuation of intracellular AMP/ATP ration [150], has been reported to be activated by noise-induced ATP depletion and responsible for the pathogenesis of NIHL [44]. Parallel to transient energy exhaustion, AMPK activates Rac-1, belonging to GTPase family which can phosphate its downstream targets via hydrolyzing GTP and trigger p67 protein phosphorylation [151]. p67 is one of the functional subunits of NADPH oxidase 3 (NOX3), which contributes to noise overexposure-induced ROS overproduction in hair cells and eventual cell death [152]. Reversely, excessive ROS accumulation also activates the AMPK*α* signaling, marked by enhanced phosphorylated AMPK*α*, while systematical administration of antioxidant N-acetyl-L-cysteine (NAC) decreases both phosphorylated AMPK*α* and oxidative stress [153]. Noise-induced phosphorylation of AMPK*α* is also regulated by liver kinase B1 (LKB1), mediated by the transient intracellular ATP depletion. Indirect inhibition of AMPK*α* or its upstream regulatory gene LKB1 can ameliorate noise-induced necrosis of acoustic hair cells [147].

However, there is hardly any effective way to boost energy generation in hair cells after noise exposure. It is reported that transient increasing serum glucose level with administration of glucose can strengthen the cochlear defense system against NIHL by increasing ATP and NADPH content in hair cells [154]. Nevertheless, the diabetic mice with constant high blood glucose show much more severe noise-induced hair cell death and spiral ganglion neuron loss, which might be due to the blood flow ration reduction induced by chronic inflammation [155].

### 3.7. Others

#### 3.7.1. Matrix Metalloproteinases

Matrix metalloproteinases (MMPs) play a key role in the extracellular matrix (ECM) homeostasis and remodeling. For example, MMP-2 is reported to be involved in synaptic remodeling after cochlear lesion [156]. Either overactivation or excessive inhibition of MMPs can lead to multiple disorders like carcinoma invasion and migration [157]. RNA sequencing data reveal that MMPs are upregulated in the acute phase after noise exposure and decreased gradually over time. A short-term administration of doxycycline, an inhibitor of MMPs, can attenuate noise-induced hearing loss while a seven-day application regimen exacerbates the hair cell death reversely [158]. MMPs are regulated by cysteinyl leukotriene and its type 1 receptor (CysLTR1), which is also increased in the cochlea after acoustic overstimulation. A four-day application of montelukast, a leukotriene receptor antagonist, can ameliorate NIHL damage [159].

#### 3.7.2. Pannexins

Like the well-researched connexin channels, pannexin protein is also involved in the intracellular communication channel formation allowing the passage of the small molecules and ions. Panx3 is one of the main kinds of pannexin protein expressed in the cochlea [160]. A previous study [161] has proved that genetic depletion of paxn3 has no impact on the proper morphogenesis of the inner ear and hearing though the cochlear bone is slightly impaired. Strikingly, the panx3 knockout mice show decreased susceptibility to NIHL while the mechanism underlying its acoustic protective effect still requires further exploration.

## 4. Gene Variations Related to NIHL

Up to now, there are more than 30 gene variations reported to be relevant to NIHL in animal studies or human epidemiology investigations.

### 4.1. Oxidative Stress and DNA Repair-Related Gene Variations

Since oxidative stress contributes to the pathogenesis of NIHL, mutations of multiple genes involving in the antioxidative system have been proved to alter noise susceptibility. Catalase (CAT), superoxide dismutase (SOD2), and the antioxidant paraoxonase (PON) are three main proteins playing important roles in scavenging free radicals; mutation of which weakens the intracellular defensive ability against noise-induced oxidative damage [71, 162, 163]. NFL2E2 (also known as Nrf2) is a vital transcriptional factor regulating various antioxidative protein expressions such as HO-1, CAT, SOD, and GPx [65]. Polymorphisms of NFL2E2 (rs6726395 and rs77684420) have been confirmed as related to genetic vulnerability for NIHL in a Chinese population-based study [164]. Other genes, like NOX3 encoding NADPH oxidase-3 catalyzing the generation of superoxide [165], HSPA1L encoding chaperons assisting antioxidative proteins to fold correctly [166], and GST (glutathione S-transferase) family [167, 168] which participate in the metabolism of an intracellular antioxidant GSH, have been reported as NIHL-related genes.

Moreover, free radicals and lipid peroxide induced by acoustic trauma could do harm to DNA integrity and might cause mutations of certain sequences. APE1 [169] and OGG1 [170], encoding DNA repair enzymes to remove specific abnormal bases, are found to be associated with NIHL susceptibility as well.

### 4.2. Apoptosis-Related Gene Variations

Another case-control study [171] involving Chinese workers with or without noise exposure history identifies two SNPs of CASP3 (rs1049216 and rs6948) significantly correlated to decreased NIHL risk, due to impaired apoptosis of hair cells since caspase3 serves as the central role of apoptosis induction.

### 4.3. Gap Junction-Related Gene Variations

Connexin 26, which is encoded by GJB2, is involved in the gap junction establishment between hair cells, allowing intercellular communications of ions and other small molecules [172, 173]. GJB2 SNP (rs3751385) is identified as a NIHL susceptible genetic mutation in a study applied in Polish workers [174]. Meanwhile, another study including Chinese workers reports that GJB2 SNP (rs137852540) combined with SOD2 and CAT mutations increased the vulnerability to NIHL [162]. The correlation between GJB mutation and NIHL vulnerability is also confirmed in Cx26 knockdown mice [174].

### 4.4. Potassium Channel-Related Gene Variations

Gene polymorphisms associated with K ion circulation in the inner ear are also associated with resistance to noise-induced hearing loss. KCNQ1/KCNE1 variation affecting potassium recycling is identified related to NIHL [175, 176]. Mutations of other potassium channel-related genes like KCNQ4 [175], KCNJ10 [176], and KCNMA1 [177] are also related to NIHL vulnerability. Potassium channels located in the outer hair cells in the cochlea which are responsible for the K^+^ influx regulate the excitability of OHCs. Reportedly, the potassium concentration in the cochlear endolymph of the guinea pig is upregulated shortly after acoustic injury [141, 178]. However, the exact mechanism behind potassium circulation disruption and NIHL is still lack of strong evidence.

### 4.5. Tip-Link-Related Gene Variations

The maturation of stereocilia in the hair cells is vital for hearing onset because it is where mechanical signals of sound waves are transformed into electrical signals that could be transported through hair cells and neurons. Tip-link, made of cadherins, plays an important part in the maintenance of ordered structure of stereocilia. A study revealed that mutations of PCDH15 and CDH23, two genes encoding cadherins for the integrity of stereocilia especially tip-link, are related to susceptibility to NIHL [179, 180].

### 4.6. Others

The purinergic receptor 2 (P2X2 receptor) is highly expressed in the cochlear sensory epithelium, especially in supporting cells of the greater epithelial ridge. P2X2 receptor can be activated by ATP [181] which is released from hair cells through connexins, and plays an important role in the development of the cochlea and hearing function [182]. A mutation of P2X2 gene (c.178G>T) is found responsible for progressive hearing loss and NIHL susceptibility in a Chinese family [183].

Another genome-wide association study suggested that AUTS2 SNP (rs35075890) and PTPRN2 SNP (rs10081191) are associated with NIHL vulnerability [184], which are commonly thought to be related to mental disorders and metabolic diseases according to previous studies [185].

There are quite more genes, with multiple physiological functions, reported to be NIHL-associated genetic factors, while the exact roles of which in the pathogenesis of NIHL still require deeper explorations (see more details in Table 1).

## 5. Therapeutics

It is widely acknowledged the lack of approved medications for NIHL treatment, and recent pharmaceutical explorations are heralding a new era in the field.

### 5.1. Approved Medications

Clinically, few medications have been identified as effective except glucocorticoid for NIHL. Glucocorticoid has been proved to exhibit various protective effects against NIHL, highlighted by its anti-inflammatory activity via inhibiting the synthesis and release of inflammatory molecules like prostaglandins and leukotrienes. Dexamethasone, as the most commonly used glucocorticoid in clinical practice, was administered postintense white noise with intratympanic or intraperitoneal injection. And two routines of administration show similar hearing recovery effects; both are significantly better than the saline group [194].

### 5.2. Drugs in Clinical Trials

The phase 2 trial report of ebselen has assessed its safety and efficacy on NIHL. Ebselen, the GPx1 mimic, aims at oxidative stress reduction. In this randomized, double-blind, placebo-controlled phase 2 trial, subjects receiving 400 mg ebselen show significantly protective effect on transient noise-induced hearing impairment with no side effects reported [195].

### 5.3. Potential Medications

#### 5.3.1. Medications Targeting Oxidative Stress

Qter, the artificial analog of an endogenous antioxidant coenzyme Q10, was administered systematically to noise-exposed mice and significantly reduced the threshold shift after acoustic trauma and promoted outer hair cell survival [196]. Moreover, the dendrites of spiral ganglion cells and cortical neuronal morphology are significantly restored by Qter application for its role involved in free radicals scavenging and regeneration of antioxidants like reduced glutathione (GSH)[197]. And redox homeostasis is maintained by Qter treatment demonstrated by reduction of 4-HNE or DHE (the markers of lipid peroxidation) immunostaining-positive cells in the cochleae.

Parallel to Qter, a peptide derived from human telomerase called GV1001 attenuates sensory hair cell death targeting the noise-induced excessive ROS/RNS accumulation, as suggested by the byproducts of lipid peroxidation (4-HNE) and protein nitration (3-NT) [58]. Moreover, GV1001 can also ameliorate kanamycin-induced hearing loss in mice, indicating that excessive ROS/RNS and its downstream cascades like inflammation and apoptosis underlie the sharing pathological contributors for noise and aminoglycoside-induced ototoxicity. Taking into consideration previous studies regarding the therapeutic effects of GV1001, we hypothesize that GV1001 might also exert otoprotective effects against NIHL, because excessive ROS/RNS generation is thought to underlie the causative mechanisms of NIHL.

The systematical administration of methylene blue prior to noise trauma promotes the acoustic hair cell survival via ameliorating the oxidative stress-induced dysfunction of respiratory electron transport chain, featured by the reservation of complex IV activity and ATP production [198]. Moreover, methylene blue induces generation of neurotrophin-3 (NT-3) to preserve the synaptic ribbons around inner hair cells upon noise exposure [199].

#### 5.3.2. Medications Targeting Inflammation

Avenanthramide-C (AVN-C), a natural flavonoid purified from oats, has shown anti-inflammatory and antioxidant activities in *in vitro* experiments. Notably, AVN-C has great water solubility and blood-labyrinth barrier permeability, suggesting pharmaceutical potential for NIHL treatment. Researchers have found that peritoneal injection of AVN-C protects hair cells from noise trauma by increasing antioxidant defense and decreasing proinflammatory cytokines like IL-1*β* and Tnf [200].

### 5.4. Medications Targeting Hearing Protective Genes

Isl1, which is highly expressed in prosensory region during otocyst development and no longer expressed in the postnatal cochlea, attenuates noise-triggered hair cell loss when artificially overexpressed specifically in postnatal hair cells. Though the exact mechanism behind the protective effect of Isl1 is not fully understood, it proposes a bold hypothesis that overexpression of acoustic progenitor developmental genes in the adult cochlea might increase the hair cell defense system against noise stress [201].

### 5.5. Emerging Nanoparticle Medications

Nanomedication, as a newly emerged therapy, was applied in the NIHL treatment. Nanosystems, including polyethylene glycol- (PEG-) coated poly lactic acid (PLA) nanoparticles and zeolitic imidazolate nanoparticles, were invented and applied to deliver steroid drugs to the inner ear. They show significant protective effect, even better than free steroid drugs, when administered systematically with better stability and biocompatibility [202, 203]. However, the efficacy and safety of nanoparticle-based medications still need further verification.

## 6. Conclusions

In this article, we systematically and comprehensively reviewed the epidemiology, mechanical and molecular pathogenesis, and therapeutic inventions about NIHL. Oxidative stress, inflammation, calcium overload, glutamate excitotoxicity, and energy metabolism are the main contributors to NIHL up to now. These pharmaceutical exploring studies not only shed light on the clinical treatment for NIHL but also make further steps on the systematical understanding of NIHL pathogenesis.

Controversies are engendered on certain disputations of NIHL: Firstly, the oxidative stress in supporting cells upon acoustic trauma has been verified, but how to promote hair cell survival through regulating supporting cells is still blank. Secondly, the excitotoxicity of excessive glutamate exacerbates the destruction of acoustic nerve while Vglut3 knockout mice show impaired neural fiber recovery. Similarly, administration of BDNF ameliorates NIHL, but depletion of Bdnf receptor gene also shows protective effect by inhibiting glutamate release. Therefore, given the complexity of glutamate and neurotrophins, what is the proper way of modulating glutamate and neurotrophins for the maximum protective effect? Thirdly, because of the blood-perilymph barrier, most medications administered systematically are hard to penetrate into the cochlea. The development of nanomedication would alter the stability and permeability of drugs, which might bring hope to the emergency of more efficacious and less toxic medication. Moreover, with the research progresses in the field of regeneration of cochlear hair cells and neural fibers [24, 204–208], NIHL might be curable one day in the future.

## Figures and Tables

**Table 1:** NIHL-related gene variations.

Class	Gene name		Variations	Function
Oxidative stress	NOX3	NADPH oxidase-3	rs33652818 [165]	Generation of superoxide (O_2_·-)
	CAT	Catalase	rs7943316 [162, 163]	Hydrogen peroxide decomposition
	GSTM1	Glutathione S-transferase mu 1	Null [167, 168]	GSH metabolism
	GSTP1	Glutathione S-transferase pi 1	Ile (105)/Ile (105) [167, 168]	GSH metabolism
	GSTT1	Glutathione S-transferase theta 1	Null [167, 168]	GSH metabolism
	NFL2E2	Nuclear factor erythroid-derived 2-like 2	rs6726395 rs77684420 [164]	Transcriptional factor regulating various antioxidative protein expression
	HSPA1L	Heat shock protein family A member 1 like	rs2227956 [166]	Antioxidative protein stabilizer
	PON2	Paraoxonase 2	S311C [71]	Antioxidative protein
	SOD2	Superoxide dismutase 2	IVS3-23T/G IVS3-60T/G [71]	Catalyzing the destruction of free radical

Apoptosis	CASP3	Caspase3	rs1049216 rs6948 [171]	Intracellular apoptosis promoter

DNA methylation	DNMT1	DNA methyltransferase 1	rs2228611 [186]	DNA methylation
	DNMT3A	DNA methyltransferase 3 alpha	rs749131 [186]	DNA methylation

Gap junction	GJB1	Gap junction protein beta1	rs1997625 [176]	Gap junction channels
	GJB2	Gap junction protein beta2	rs3751385 [162, 176]	Gap junction channels
	GJB4	Gap junction protein beta4	rs755931 [176]	Gap junction channels

DNA repair	APE1	Apurinic/apyrimidinic endodeoxy-ribonuclease 1	T1349G T656G [169]	Encoding the endonuclease, a DNA repair enzyme
	OGG1	8-Oxoguanine DNA glycosylase	Ser326Cys [170]	DNA repair enzyme

Ion channels	KCNMA1	Potassium calcium-activated channel subfamily M alpha1	rs696211 [177]	Ca^2+^-activated K^+^ channel
	KCNE1	Potassium voltage-gated channel subfamily E regulatory subunit 1	rs2070358 rs1805128 [175, 176]	K^+^ channel
	KCNJ10	Potassium inwardly-rectifying channel subfamily J member 10	rs1130183 [176]	K^+^ channel
	KCNQ1	Potassium voltage-gated channel subfamily Q member 1	rs7945327 rs11022922 rs718579 rs163171 rs2056892 [175, 176]	K^+^ channel
	KCNQ4	Potassium voltage-gated channel subfamily Q member 4	rs4660468 [175]	K^+^ channel

Stereocilia structure	PCDH15	Protocadherin related 15	rs11004085 rs7095441 [179]	Encoding cadherin for the integrity of stereocilia, especially tip-link
	CDH23	Cadherin 23	rs3752752 [180]	Encoding cadherin for the integrity of stereocilia, especially tip-link
	MYH14	Myosin heavy chain 14	rs667907 rs588035 [179]	Might contribute to tip-link integrity

Others	EYA4	EYA transcriptional coactivator and phosphatase 4	rs3813346 [187]	Multifunctional transcriptional factor
	FOXO3	Forkhead box O3	rs2802292 rs3777781 rs212769 [188]	Multifunctional transcriptional factor
	GRHL2	Grainyhead-like 2	rs611419 [189, 190]	Multifunctional transcriptional factor
	HOTAIR	HOX transcript antisense RNA	rs4759314 [191]	Regulatory lncRNA
	MYO1A	Myosin 1a	rs1552245 [177]	Actin-based molecular motors
	NOTCH1	Notch receptor 1	rs3124594 rs3124603 [192]	Notch signaling transduction
	NCL	Nucleolin	rs7598759 [193]	Ribosome biogenesis
	P2RX2	Purinergic receptor P2X2	V60L [183]	Purinergic signaling transduction
	AUTS2	Autism susceptibility candidate 2	rs35075890 [185]	Multifunctions
	PTPRN2	Protein tyrosine phosphatase receptor type N2	rs10081191 [184]	Multifunctions
